# A Facile, One-Pot, Surfactant-Free Nanoprecipitation Method for the Preparation of Nanogels from Polyglycerol–Drug Conjugates that Can Be Freely Assembled for Combination Therapy Applications

**DOI:** 10.3390/polym10040398

**Published:** 2018-04-03

**Authors:** Laura I. Vossen, Stefanie Wedepohl, Marcelo Calderón

**Affiliations:** Freie Universität Berlin, Institut für Chemie und Biochemie, Takustrasse 3, 14195 Berlin, Germany; laura.vossen@fu-berlin.de (L.I.V.); stefanie.wedepohl@fu-berlin.de (S.W.)

**Keywords:** combination therapy, prodrug, polyglycerol, nanogel, doxorubicin, paclitaxel

## Abstract

A well-established strategy to treat drug resistance is the use of multiple therapeutics. Polymer-based drug delivery systems (DDS) can facilitate a simultaneous delivery of two or more drugs. In this study, we developed and synthesized a dendritic polyglycerol (PG) nanogel (NG) system that allows for free combination of different fixed ratios of active compound conjugates within a single NG particle. As a proof of concept, we synthesized NGs bearing the chemotherapeutic agent doxorubicin (DOX) and paclitaxel (PTX) in different ratios, as well as conjugated dye molecules. Our combination PG NGs were formed by simply mixing PG–drug/dye conjugates bearing free thiol groups with PG-acrylate in an inverse surfactant-free nanoprecipitation method. With this method we obtained PG-NGs in the size range of 110–165 nm with low polydispersity indices. Solubility of hydrophobic PTX was improved without the need for additional solubilizing agents such as polyethylene glycol (PEG). Interestingly, we found that our NGs made from PG-DOX conjugates have a high quenching efficiency for DOX, which could be interesting for theranostic purposes.

## 1. Introduction

Combination therapy plays a key role in cancer treatment. However, the use of most anticancer drugs is limited by toxicity and the development of drug resistance. This is due to the inability of an administered drug cocktail to achieve the appropriate spatiotemporal distribution [1,2,3,4].

Consequently, to fulfill effective delivery of multiple therapeutics to the right place at the right time, polymer-based combination therapy systems have been designed and investigated [5]. Such drug delivery systems (DDS) should improve the pharmacokinetics and efficacy of bioactive agents while reducing their side effects. DDS can facilitate the co-delivery of two or more drugs at the target site and hence a decreased dosage of the drugs is needed, side effects reduced, and drug resistance possibly overcome [6,7]. Two simultaneously administered drugs can undergo synergistic, additive, and potentiation mechanisms [8]. A common approach is to load the drugs to a single nanocarrier in order to achieve a greater combinatorial effect than the sum of individual drugs, hence a pharmacodynamically synergistic combination [6]. Moreover, the design of DDS that are responsive to different stimuli such as pH, temperature, and redox conditions will provide a defined release of the drugs at the target site. pH-sensitiveness can be exploited for instance for a triggered drug release at slightly more acidic pH of tumors and/or more acidic cellular compartments (endosomes and lysosomes) [9,10].

A variety of macromolecules have been proposed for the preparation of DDS, among which dendritic polyglycerol (PG) has therapeutic relevance for biomedical applications due to many exceptional properties such as structure, easy and controlled preparation to the kilogram scale, easy tunable functional groups for attaching biologically active molecules, and finally biocompatibility and water solubility [11,12].

One method to achieve a DDS in the nanometer range is the synthesis of nanogels (NGs) [13]. NGs are crosslinked polymers forming networks that fill the size gap between dendrimers/polymers (e.g., polymer–drug conjugates) with diameters of 10–20 nm and microscopic hydrogels [14]. NGs are of interest in biomedical applications since they have similar dimensions as biomacromolecules including viruses and proteins. Advantages of NGs over other drug delivery systems are mainly due to their soft nature, large water content, high loading capacity, and colloidal stability. They can be prepared via mini- [15,16,17] and micro-emulsion [18] but in this case high energy input because ultrasonication or large amounts of surfactants are needed to stabilize the droplet reactors. To avoid such harsh conditions, surfactant-free nanoprecipitation techniques have been developed [19,20].

Several preparations of NGs using PG have been reported by the Haag and Calderón groups [21,22,23,24,25,26,27,28] and encapsulation of bioactive molecules including anticancer drugs into PG NGs as well as their in vitro release has been extensively investigated. However, encapsulation can cause unwanted leakage and release of the payload before reaching the target site. Furthermore, encapsulation of hydrophobic drugs, for instance paclitaxel (PTX), into a hydrophilic PG core will only be possible through copolymerization with another hydrophobic polymer. Co-encapsulation of both hydrophilic and hydrophobic drugs presents the same difficulties. However, covalently bound bioactive molecules can gain solubility and prevent leakage, and when attached via a degradable bond allow for a controlled release of the drug at specific trigger conditions.

In our group, we have established the synthesis of PEGylated PG conjugates combining two anticancer drugs, doxorubicin (DOX) and PTX, on a single molecule which showed promising results in vivo [29]. Those conjugates typically aggregate in water and buffer, which lead to a mixture of different conjugates rather than to one defined PG–drug conjugate. In this case PEGylation served as a solubilizing and size-increasing agent, but since PEG has already been controversially discussed due to its unfavorable immune responses and other side effects, research towards PEG alternatives is being performed [30].

Since functionalized PG-conjugates can serve as building blocks for the synthesis of NGs, individual PG–drug conjugates could be easily combined into NGs that serve as DDS containing, for example, two different therapeutic agents in a known synergistic ratio, a drug and an imaging agent, or virtually any conceivable combination of conjugated functional moieties on a single carrier. The formation of a PG NG would therefore abolish the requirement for PEG, since the NG itself retains a large amount of water and therefore enhances the solubility of a drug.

**Figure 1:**
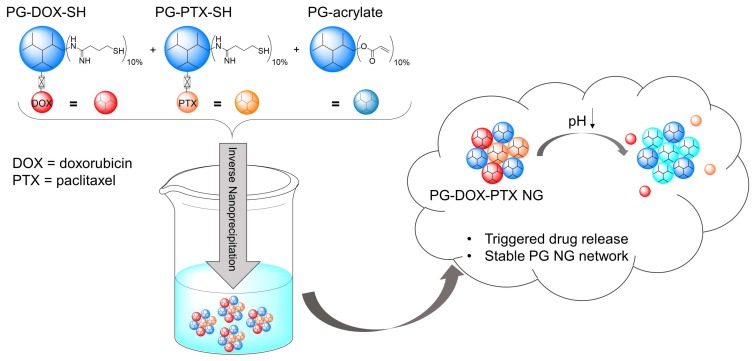
Preparation of multidrug PG NGs by an inverse nanoprecipitation method and their behavior in acidic pH. PG–drug conjugates were functionalized with approximately 10% free thiol groups (SH) in order to react with 10% acryl groups on the macro crosslinker PG-acrylate.

In this study, we aimed to find a method for a mild PG NG preparation achieving combination-DDS of two anticancer drugs, DOX and PTX, as well as drug/dye combinations. The NG should be prepared by a simple PG–drug/dye conjugate crosslinking approach where the functional moieties should be conjugated before NG preparation to PG through a cleavable hydrazone bond in order to avoid leakage from the scaffold and achieve a triggered drug release. We present a surfactant-free one-pot approach for the preparation of a freely combinable PG NG system (Figure 1). Our synthetic conditions could be applied to PG NGs carrying only one drug or a dye as well as to NGs combining two drugs or a drug and a dye, showing the versatility at different combinations of the system. We present the characterization of this variety of NGs regarding their size, structure, release properties, and stability.

## 2. Experimental Section

### 2.1. Materials

Solvents were purchased from Acros Organics (Berlin, Germany) and used as received unless stated otherwise. Doxorubicin hydrochloride (DOX) and Paclitaxel (PTX) were purchased from Yick-Vic Chemicals & Pharmaceuticals (Hong Kong, China) and further the hydrazone derivative of DOX (DOX-EMCH) as well as the hydrazone derivative of paclitaxel (PTX-EMCH) and the non-cleavable control DOX-mal were prepared as described previously [31,32,33]. The indodicarbocyanine maleimide dye (IDCC-mal) was obtained from Epiios Therapeutics GmbH (Berlin, Germany). Dendritic polyglycerol (PG) (average MW 10 kDa, PolyDispersity Index (PDI) = 1.6, approximately 135 OH groups) was prepared according to published procedures [34]. Dialysis tubes with a molecular weight cut-off (MWCO) of 50 kDa were obtained from Spectrum (München, Germany). Amicon Ultra-15 centrifugal filters (MWCO 3 kDa) were purchased from Merck Millipore (Darmstadt, Germany). Sephadex G-25 superfine was obtained from GE Healthcare (München, Germany). Size exclusion chromatography (SEC) of PG–drug/dye conjugates was performed with Sephadex G-25 superfine under ambient pressure and room temperature (rt).

### 2.2. Instrumentation

FT-IR analysis was carried out using a JASCO FT-IR 4100 LE spectrophotometer. ^1^H-NMR spectra were recorded on a Jeol ECX 400 or on a Bruker AMX 500. Chemical shifts are reported in ppm (δ units). NMR spectra were analysed with MestreNova software. The fluorescence spectra were recorded on a Jasco FP-6500 spectrofluorometer. UV/Vis spectra were recorded on a Scinco S-3100 spectrophotometer. Reverse phase-HPLC (RP-HPLC) and gel permeation chromatography (GPC) were performed on a Shimadzu Prominence-I LC-2030 liquid chromatography system with an internal UV absorption detector and a Shimadzu RID-29A refractive index detector. Deionized water (MilliQ water) (resistivity ~18 MΩ cm^−1^, pH 5.6 ± 0.2) was used in all experiments and for preparation of all samples. If not otherwise specified, phosphate buffer (PB) (50 mM) was used for the pH of 7.4, and acidic pH values were reached with a sodium acetate buffer (pH 4.0, 50 mM). All measurements were carried out with freshly prepared solutions at 25 °C. pH values were measured with a Scott instruments HandyLab pH meter at 25 °C. Zeta potential and dynamic light scattering (DLS) measurements were performed using a Malvern Zetasizer Nano-ZS 90 (Malvern Instruments, Malvern, UK), equipped with an integrated He–Ne laser (at λ = 633 nm or 532 nm). DLS measurements were performed at 25 °C under a scattering angle of 173° using quartz cells. Surface charge measurements were performed at 25 °C using folded capillary cells (DTS 1070). Prior to all measurements, NG samples (1 mg mL^−1^ in MilliQ water) were filtered through a cellulose acetate (CA) membrane filter with 0.45 μm pore size. Transmission electron microscopy (TEM) was performed on a Hitachi Scanning Electron Microscope (SU8030, Hitachi High-Technologies Corporation, Tokyo, Japan) (20 kV) at different magnifications. For preparation, one droplet (2 µL) of NG solution (1 mg mL^−1^ in MilliQ water) was attached onto a carbon-coated copper grid and dried by air followed by a droplet (5 µL) of uranyl acetate (1%). The excess contrasting material was removed by means of filter paper and the sample could dry in air.

### 2.3. Synthesis of PG-Acrylate

PG-acrylate (10% acrylation) was obtained following a procedure previously reported [35]. Briefly, PG was dissolved in DMF and triethylamine was added. The solution was cooled down to 0 °C and a solution of acryloyl chloride was slowly added in DMF. The solution was stirred for 12 h at rt. Finally, the precipitate was removed by filtration and the solution was dialyzed (MWCO 2 kDa) in CHCl_3_ for three days and then against water for another two days. The product was stored at 4 °C in MilliQ water and stabilized with 1000 ppm of the radical scavenger 4-methoxyphenol. The acrylation degree was determined from the ^1^H-NMR. ^1^H-NMR (400 MHz, D_2_O): 6.48–6.51 (m, 0.12 H, CH*_2_*=CH-), 6.24–6.29 (m, 0.12 H, CH_2_=CH), 6.06 (s, 0.12 H, CH*_2_*=CH-), 3.66–4.32 (m, 5 H, PG).

### 2.4. Synthesis of PG-Amine

PG-amine (10% amination) was synthesized according to previously reported methodologies [34,36]. Briefly, PG-amine was prepared by a three-step protocol starting from PG and a conversion of 12% of the OH groups into mesyl groups, followed by transformation of the mesyl groups into azide functionalities, and finally reduction of the azide groups to amine groups by using triphenylphosphine as reducing agent. Extensive dialysis was carried out after each reaction step and quantification of the NH_2_ groups was performed using ^1^H-NMR spectroscopy. The product was stored at 4 °C in methanol. ^1^H-NMR (400 MHz, D_2_O): 3.94–3.54 (m, 5 H, PG), 3.02–2.84 (m, 0.12 H, NH_2_).

### 2.5. Synthesis of PG–Drug/Dye–SH Conjugates

PG-amine (*M*_n_ ≈ 10 kDa, 10% amine groups, 20 mg, 0.002 mmol, 13.51 NH_2_ groups) was dissolved in 0.3 mL MilliQ water. 2-iminothiolane (5.6 mg, 0.04 mmol, 1.5 eq per NH_2_ group) was dissolved in 0.15 mL MilliQ water and added to the solution. The reaction mixture was stirred for 20 min at rt. Afterwards, the reaction mixture was directly applied on a Sephadex-G25 column and eluted with MilliQ. The polymeric fraction (PG-thiol: PG-SH) was collected and to this solution 1.0 eq per PG of a prodrug (DOX-EMCH, DOX-mal, or PTX-EMCH) or a dye (IDCC-mal or fluorescein isothiocyanate: FITC) was added in DMF (0.1 mL) and stirred for 2 h. Afterwards, the PG–drug/dye solution was concentrated and washed with MilliQ water using Amicon centrifugal filters (MWCO 3 kDa) and in the end purified by SEC using a Sephadex-G25 superfine column eluting with MilliQ water. The obtained yields were between 75–80%. All PG–drug/dye conjugates were kept at 4 °C with catalytic amounts of tris(2-carboxyethyl) phosphine hydrochloride (TCEP). Successful coupling reaction was monitored by an Ellman’s test where the thiol concentration was followed via UV/Vis-Spectroscopy at 412 nm [37]. Drug and dye loading on PG-DOX-SH, PG-DOX(non)-SH, PG-PTX-SH, PG-IDCC-SH, and PG-FITC-SH conjugate were determined by UV/Vis-Spectrometry using the molar absorption coefficient of IDCC at 650 nm = 208,000 M^−1^ cm^−1^, of DOX at 495 nm = 10,645 M^−1^ cm^−^^1^ and FITC at 500 nm = 80,000 M^−1^ cm^−1^. The amount of bound PTX on PG-PTX-SH conjugate was determined by ^1^H-NMR spectroscopy (Figure S1) as described in earlier publications [38,39]. The average drug/or dye-to-polymer ratio was approximately 1 drug/or dye molecule per polymer.

### 2.6. General Procedure for the Preparation of PG-NGs

PG-NGs were obtained following an adapted previously reported procedure [24]. PG-acrylate (10% acrylation) (2.5 mg) and PG–drug/dye–SH (10% thiolation) (2.5 mg) were diluted separately in 1 mL MilliQ water and cooled down to 4 °C. The two macromonomers were mixed at 4 °C and the mixture (5 mg/2 mL) was immediately precipitated in 40 mL acetone (stirred in a 100 mL-flask at 900 RPM). Shortly after, the stirrer was removed, and the reaction mixture stood for two days at rt, when the reaction between thiol and acrylate groups could take place in a Michael-type addition. The reaction was quenched with 2-hydroxethyl acrylate. After two days, 3 mL MilliQ water was added to the solution and acetone was evaporated. The nanogels were dialyzed (50 kDa MWCO) for 3 d in Milli-Q water and afterwards kept in solution at 4 °C. Resulting NGs were kept at a concentration of 0.5 mg/mL. The obtained yields were between 85–90%.

In the case that three macromonomers were used in the reaction, the concentration of PG-acrylate (10%) stayed the same (2.5 mg) and the sum of used PG–drug/dye–SH (10%) conjugate should be added to 2.5 mg. (Example: 2.5 mg PG-acrylate + 1.25 mg PG–drug–SH + 1.25 mg PG–drug–SH).

Drug and dye loadings in weight percent (w%) were determined by UV/Vis-Spectrometry using the molar absorption coefficient of IDCC at 650 nm = 208,000 M^−1^ cm^−1^, of DOX at 495 nm = 10,645 M^−1^ cm^−1^, and FITC at 500 nm = 80,000 M^−1^ cm^−1^. The PTX w% amount was calculated theoretically according to the mean w% values of DOX incorporated into the NG scaffold after synthesis.

### 2.7. RP-HPLC Analysis of PTX Release

The PTX release from PG-DOX-PTX NG was analyzed by RP-HPLC as previously described [29,39]. The release of PTX was analyzed at 254 nm and LabSolutions software was used for evaluation. A Hypersil™ ODS C18 column (Thermo Scientific, Waltham, MA, USA, 100 mm × 4.6 mm, particle size: 5 μm) connected to a guard C18 column was employed. Acetonitrile–water (65:35) was used as the mobile phase at a flow rate of 1.0 mL min^−1^ under isocratic regime. The injection volume was 50 μL. Stock solutions of PTX in acetonitrile were prepared and assessed by RP-HPLC to obtain a calibration curve for PTX (0.5–5 μg, R = 0.999) (Retention time: 2.85 min) (Figure S2a). The release profiles were analyzed at pH 7.4 and 4.0 (Figure S2b). For that purpose, the NG (constant PTX concentration, 0.5 mg mL^−1^ NG concentration) was incubated with acetate buffer of pH 4.0 (50 mM) and PB pH 7.4 (50 mM). Samples were prepared in duplicates and maintained at 37 °C under continuous shaking. Aliquots (150 μL) were taken at different time intervals (0, 1.5, 4.5, 6.5, and 24 h), freeze-dried, reconstituted in 200 µL acetonitrile, and finally analyzed by RP-HPLC.

### 2.8. GPC Analysis of PG-DOX NG

Size exclusion chromatography was performed on a Shimadzu Prominence-i LC-2030 liquid chromatography system equipped with a Shimadzu RID-20A refractive index detector. The GFC column used was a Shodex OHpak SB-806M HQ with OHpak SB-G 6B as guard column. Milli-Q water was used as the mobile phase with a flow rate of 0.5 mL min^−1^ under isocratic regime. The injection volume was 50 µL from a NG solution of 1 mg mL^−1^ concentration.

### 2.9. Fluorescence Analysis of DOX Release

Different NGs (PG-DOX NG, PG-DOX(non) NG) and conjugate PG-DOX-SH were incubated in acetate buffer of pH 4.0 (50 mM) or PB pH 7.4 (50 mM) at 37 °C. The fluorescence was recorded exciting the samples at 500 nm, in the absorption range of the DOX, over time. Quenching efficiency was calculated with following equation:

(1) $$\mathrm{Quenching}\;\mathrm{efficiency}=100\times [1-\frac{\mathrm{fluorescence}\;\mathrm{intensity}\;\mathrm{of}\;\mathrm{the}\;\mathrm{probe}}{\mathrm{fluorescence}\;\mathrm{intensity}\;\mathrm{of}\;\mathrm{the}\;\mathrm{probe}\;\mathrm{after}\;\mathrm{cleavage}\;}]$$

## 3. Results and Discussion

### 3.1. Synthesis and Characterization of PG-DOX-PTX NGs by an Inverse Nanoprecipitation Method

Before preparation of combinatory NGs, the PG-conjugates serving as building blocks were synthesized. As model drugs with known synergistic effects we employed DOX and PTX, as we got promising results in combination therapy trials of the individual PEGylated PG-conjugates in an earlier study [29]. Drugs were covalently bound to PG via a pH-sensitive hydrazone bond in order to be releasable in acidic environment. PG–drug/dye conjugates were synthesized in a one-pot reaction as described before [29,32,40,41,42].

**Figure 2:**
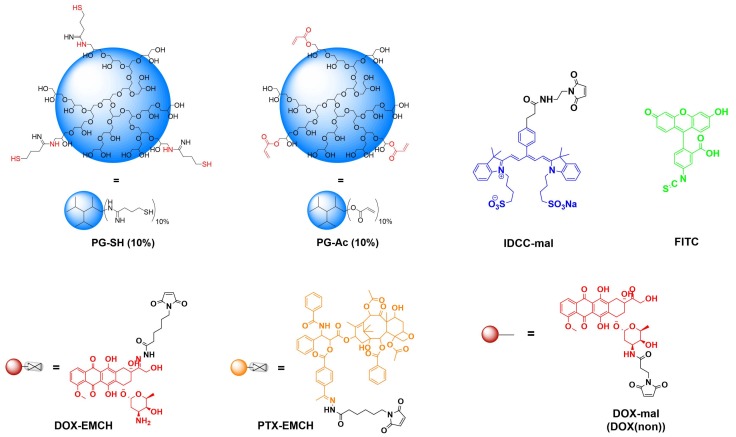
Building blocks for NG synthesis.

**Figure 3:**
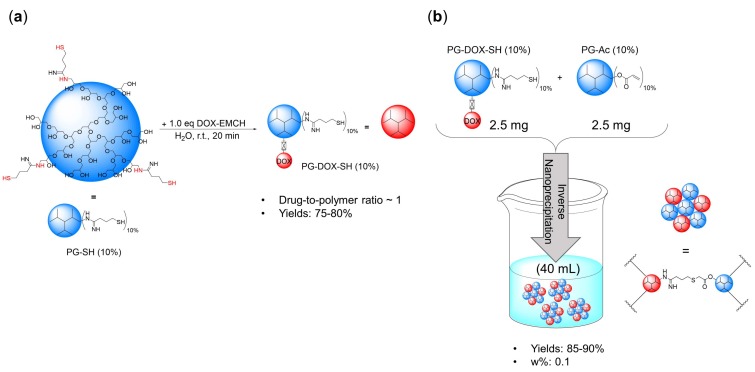
(**a**) Preparation of PG-DOX-SH conjugate and (**b**) PG-DOX NG via inverse nanoprecipitation.

The strategy for NG synthesis was adapted from M. Dimde et al. [24] and detailed structures of all used building blocks in this study are depicted in Figure 2. In general, NGs should be synthesized from PG-acrylate with 10% acrylation (PG-Ac (10%)) serving as a macro crosslinker, connecting with PG–drug/dye conjugates by an inverse nanoprecipitation method in acetone as a non-solvent. In this Michael-type addition, remaining thiol groups on the PG–drug/dye conjugates react with the acrylate groups of PG-Ac (10%), forming the cross-linked network of the NG (Figure 3).

**Figure 4:**
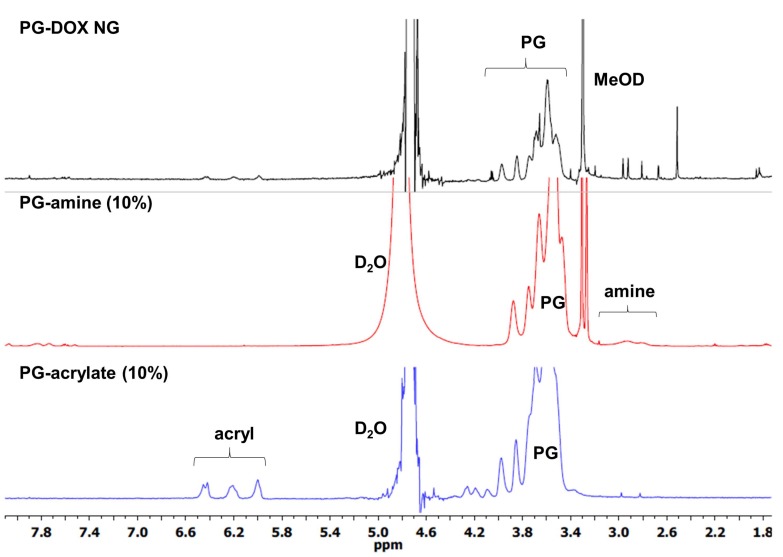
^1^H-NMR spectra of macromonomers (D_2_O) and PG-DOX NG (MeOD).

First, to establish the conditions for our NG system, PG-DOX NG was synthesized. The precursors PG-amine (PG-NH_2_ (10%)) and PG-Ac (10%) were prepared according to published procedures [16,34] and their synthesis is described in the experimental section. PG-NH_2_ (10%) was thiolated with 2-iminothiolane to obtain PG-thiol with 10% thiolation (PG-SH) (10%) and then reacted with doxorubicin 6-maleimidocaproic hydrazide (DOX-EMCH). Unreacted DOX-EMCH was removed by ultrafiltration and gel permeation chromatography. The stock solution of precursor PG-DOX-SH (10%) was kept at 4 °C with catalytic amounts of tris(2-carboxyethyl) phosphine hydrochloride (TCEP) to reduce disulfide formation of free thiol groups. Then, both macromonomers, PG-DOX-SH (10%) and PG-Ac (10%), were dissolved in water at 0 °C and precipitated in acetone under vigorous stirring. Fast diffusion of the water phase into the acetone phase caused a drastic increase of the macromolecular precursors concentration and therefore crosslinking could take place. The progress of this Michael-type addition and NG formation could be monitored by ^1^H-NMR through the significant reduction of acrylate and methacrylate peaks at 5.5–6.5 ppm (Figure 4). Resulting NGs were extensively purified by dialysis in MilliQ water and stored at 4 °C.

**Figure 5:**
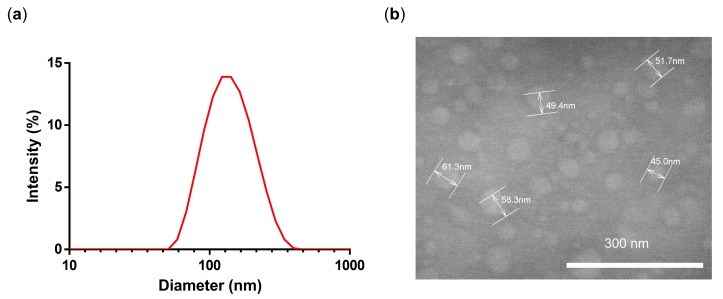
(**a**) DLS intensity size distribution and (**b**) TEM of NG3.

The reaction conditions screened for synthesizing PG-DOX NGs are listed in Table 1. The hydrodynamic diameter and polydispersity index (PDI) was analyzed by DLS (Figure 5a). With this nanoprecipitation method we could achieve sizes in the range of 133 to 210 nm. We chose to work with the conditions of NG3 for the preparation of our NG combining DOX and PTX since we assumed the smallest size of 133 nm with a PDI of 0.14 would be most suitable for systemic administration.

NG3 was characterized by TEM in order to analyze the shape and the size of the NG. TEM images showed the presence of spherical, uniformly distributed particles with a mean diameter of 50 nm (Figure 5b). The smaller diameter in TEM can be attributed to partial deswelling of the particles on the carbon-coated copper grid, as previously reported for other hydrophilic NGs [23]. Furthermore, according to a gel permeation chromatography results, we could verify that there is no free drug since there is only one peak at 488 nm (Figure S3).

**Figure 6:**
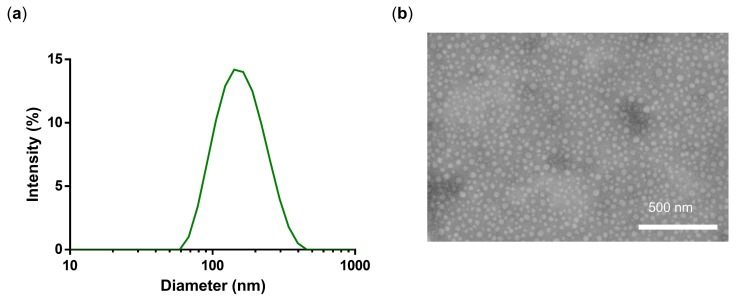
(**a**) DLS intensity size distribution and (**b**) TEM of NG4.

The found synthetic conditions (NG3) were then applied to the synthesis of PG-DOX-PTX NGs of different composition (Table 2). In this case, three macromonomers, PG-DOX-SH, PG-PTX-SH, and PG-Ac, were mixed and dissolved in MilliQ water at 0 °C and precipitated in acetone. The concentration of PG-Ac was constant (2.5 mg/mL) and the concentration of both PG–drug conjugates added to 2.5 mg/mL in order to have a total macromonomer concentration of 5 mg/2 mL. Here it is worth mentioning that the NG synthesis also worked when both drugs were previously conjugated to a single PG nanocarrier (Table S1). However, small sizes of 110–150 nm and small PDIs could only be achieved by pre-synthesizing two distinct PG–drug conjugates and incorporating them separately into the NG system (Table 2). We show that conjugation of two drugs to a single PG is limited to solubility and sterical issues as reported in the literature [29]. Hence, our approach combining several PG-conjugates into a NG with one active compound is controllable and reproducible. Hydrodynamic diameters of synthesized PG-DOX-PTX-NGs listed in Table 2 were between 110–148 nm (Figure 6a, NG4) and TEM images revealed a spherical size and a shrunken diameter of around 30–40 nm (Figure 6b), similarly to what we observed for NG3. This means that our selected conditions of NG3 were applicable to a more complex NG system combining two chemotherapeutic drugs and even allowed us to control the drug ratio (Table 2). Drug loadings in weight percent (w%) were 0.5:0.9 for a 1:1 DOX to PTX ratio and 0.8:0.3 for a 5:1 ratio. We chose a ratio of 5 to 1 (DOX to PTX) since in the past we could show that this ratio would have a synergistic effect [29].

Regarding the hydrodynamic diameter, we expected similar sizes for all synthesized NGs with marginal differences due to the synthetic nanoprecipitation method that is performed manually and would therefore be subject to minor variations. The main difference between the two used drugs was the hydrophobicity. PTX is highly hydrophobic and barely soluble in water, compared to the more hydrophilic hydrochloride form of DOX. When the building block conjugates PG-DOX-SH and PG-PTX-SH were prepared, we assumed that aggregate formation for PG-PTX-SH conjugate was more likely and hence would have resulted in larger NGs. We did however not see any significant changes for the diameters of our NGs NG4 and NG5 (Table 2). A slightly reduced diameter could be observed for NG6 (110 nm) with a DOX to PTX ratio of 5:1. The PDIs did not show any specific trend and ranged from 0.12 to 0.23 (Table 2). In order to explore the versatility of the NG system and its potential for application for a variety of different functional moieties, we synthesized a NG with fluorescein isothiocyanate (FITC) and a NG combining DOX and an indodicarbocyanine (IDCC) dye (Table 2) using the same strategy. The hydrodynamic diameter for FITC-labeled NG (NG7) showed a size of 125 nm and similar PDI of 0.18 compared to other synthesized NGs. PG-DOX-IDCC NG (NG8) had an increased size of 164 nm and a PDI of 0.09 (Table 2). We assumed that both moieties, DOX and IDCC, were more hydrophilic than the combination of DOX and PTX and hence less aggregate formation during precursor synthesis would occur and therefore the PDI of the NG was reduced.

With this, we concluded that NGs with different PG-conjugate building blocks of various natures could be freely combined and we expected that the obtained sizes of 110–164 nm were still in the nanometer range ideal for systemic administration.

### 3.2. Stability of PG-DOX NG

**Figure 7:**
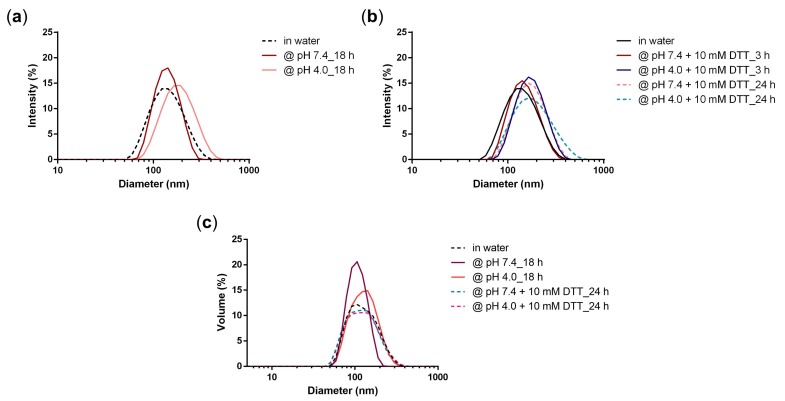
Intensity size distribution of NG3 at (**a**) pH 7.4 and pH 4.0 after 18 h and at (**b**) pH 7.4 and 4.0 in the presence of 10 mM DTT after 3 h and 24 h; (**c**) Volume size distribution of NG3 at pH 7.4 and 4.0 and in the presence of 10 mM DTT after 24 h.

The stability of the NG network was studied by DLS at different pH values (Figure 7a) and in the presence of a reductive agent dithiothreitol (DTT) to confirm that there was no disulfide formation during NG preparation (Figure 7b,c).

All DLS measurements showed that the NG network stayed stable over time (Figure 7). We expected that there could have been a marginal size change at acidic pH, due to the release of the drug, but overall the NG network should have stayed stable and no smaller constructs should be observed regarding the DLS size distributions by intensity and volume. Haag and coworkers reported a DOX prodrug NG that contained a crosslinked network through disulfide bridges [21]. In that case a degradation study with addition of 10 mM DTT measured by DLS demonstrated a significant size decrease from around 150 nm to 5 nm. In our case, when a phosphate buffer pH 7.4 (50 mM) was added to the system, the size of the NG as well as the PDI increased only to a marginal extent (148 nm PDI 0.25). When an acidic acetate buffer pH 4.0 (50 mM) is added to the system, drug release should have occurred (as demonstrated before) which caused the size of the NG to slightly increase as well, but interestingly, the PDI in this case stayed the same (152 nm, PDI 0.14). We would in this case expect a small disruption of the NG network and hence possibly an increase of the PDI, which did not happen. The addition of the reductive agent DTT at different pHs did not affect the size, however PDIs were higher in all cases (PDI 0.21–0.25). Overall, these results suggest that while releasing the drugs at acidic media, the NG network stays stable. The stability in the reductive environment additionally demonstrates that the NG network was solely formed through a Michael-type addition between thiol and acrylate groups and no disulfide bridges were formed.

### 3.3. Triggered Release of DOX and PTX from the NG

**Figure 8:**
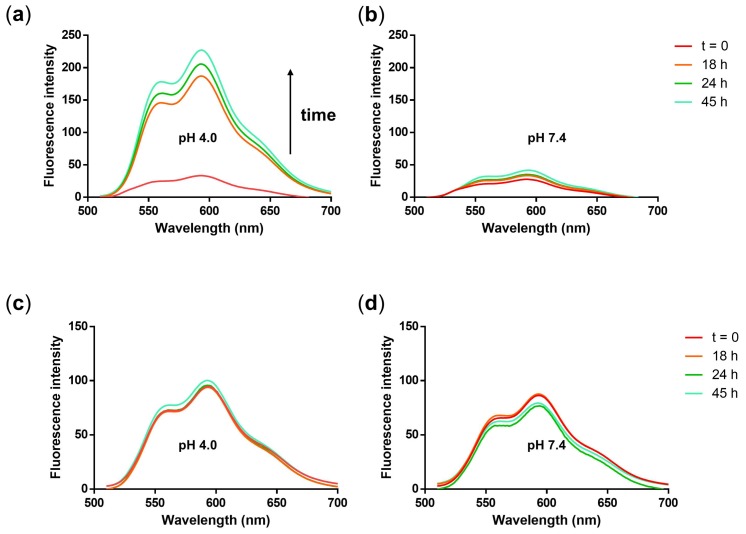
Fluorescence emission spectra of NG3 after incubation in (**a**) acetate buffer (pH 4.0, 50 mM) and (**b**) phosphate buffer (pH 7.4, 50 mM) at 37 °C over 45 h. Fluorescence emission spectra of non-cleavable PG-DOX(non) NG after incubation in (**c**) acetate buffer (pH 4.0, 50 mM), and (**d**) phosphate buffer (pH 7.4, 50 mM) at 37 °C over 45 h.

The design of our NGs includes a cleavable hydrazone bond in order to obtain a site-specific drug release at the target site, for instance in acidic tumor environment. Therefore, we analyzed the release of the drugs at 37 °C and acidic pH from the NG. DOX release was measured quantitatively at pH 4.0 and 7.4 by analyzing the fluorescence of DOX exciting the sample at 500 nm. A significant increase of DOX fluorescence was observed over a time period of 45 h at acidic pH (Figure 8a). In contrast, the DOX fluorescence increased only minimally at pH 7.4 (Figure 8b). A release profile from the fluorescence data of Figure 8a,b was plotted by taking the fluorescence intensity values at 592 nm and plotting them over time (Figure S4).

With this method, we could show a release of DOX at acidic conditions and confirmed an almost stable association of the drug with the NG at neutral pH. Notably, we observed that our NG system quenched the DOX fluorescence, as can be seen in Figure 6a, enabling us to perform the release study by simple fluorescence measurement. In fact, the quenching efficiency was even up to 86% (calculated with Equation (1)). This is in the range of similar systems reported in the literature [43]. Quenching of DOX fluorescence has proven useful for monitoring pH-labile bond cleavage in real time in cells. In that study, a PG conjugate bearing a combination of DOX and an IDCC dye as a quenching moiety on a single pH-labile linker was used as a macromolecular theranostic probe [32]. The quenching efficiency in this system was up to 94%. The observation that DOX-EMCH can be quenched by itself has been reported in the literature and appears to be due to C=N isomerization and N–N free rotation of the hydrazone linker. When activated under acidic conditions, hydrolyzation of the hydrazone bond afforded free DOX and therefore an increase of the fluorescence [43]. To confirm our interpretation of the fluorescence spectra as quenching and release, we synthesized a NG built from a PG-DOX conjugate which was not cleavable. For this, a non-cleavable prodrug DOX-mal (non) was synthesized (Figure 2) that was functionalized with maleimidopropionic acid at the daunosamine position resulting in a stable amide bond.

For PG-DOX(non) NG, no increase in DOX fluorescence at both neutral and acidic pH was observed, as well as a higher fluorescence signal at the same DOX concentration as PG-DOX NG (Figure 8c,d). For PG-DOX-SH conjugate an increase of the fluorescence signal could be observed, however the quenching efficiency in this case was only 52% (Figure S5). These results prove that the quenching properties of DOX arose from the hydrazone bond and a controlled drug release from the NG scaffold in acidic media could be achieved. Moreover, since for PG-DOX-SH conjugate the quenching efficiency was only 52%, we assumed that the method with which the NG was prepared played an important role. Our results suggest that DOX remained in the core of our NG and would therefore be hidden and protected from degradation.

The release of PTX from PG-DOX-PTX NG was studied at pH 4.0 and pH 7.4 by an RP-HPLC method (Figure S4). In accordance with previous publications, where we show a controlled PTX release from PG–drug conjugates, PTX release was enhanced at pH 4.0 and almost no release could be observed at pH 7.4 [29,38]. Hence, our *in vitro* release studies show that DOX and PTX were being released from the NG network in a controlled manner and these properties should enable both drugs being released in slightly acidic tumor environment or cellular compartments (e.g., lysosomes or endosomes) [44].

## 4. Conclusions

We have developed a simple, reproducible, one-pot surfactant-free approach that allowed the preparation of multi-drug PG NG systems. Our PG NGs had sizes between 110 and 164 nm with small PDIs (0.09–0.23). We were able to load different ratios and different drug/dye weight percentages into the NGs, which allows a precise tuning and free mixing of different PG-conjugates to form a combination PG NG. Moreover, we could demonstrate that the drugs were released in a controlled manner at acidic pH and that the NG network stayed stable under reductive and acidic conditions. Overall, our synthetic approach for the synthesis of PG NGs combining DOX and PTX could be a good platform as a nano drug delivery system for combination therapy. We have demonstrated that the synthesis can be applied to form any type of PG NG combining two chemotherapeutics or a drug and a dye. This would allow one to synthesize several different NGs and to perform a screening of the adequate drug ratio. Besides, our NGs carrying DOX showed a high quenching efficiency of the DOX fluorescence which could be suitable for a real-time drug release monitoring in cells and for obtaining detailed information of our NG’s intracellular fate. This means that our PG NGs should be further investigated for their therapeutic and theranostic properties.

## Figures and Tables

**Table 1:** PG-DOX NGs prepared at different concentrations of macromonomer solution and volume ratios between solvent and non-solvent.

#	c [Monomers]	Acetone (mL)	d [nm] Water	PDI
NG1	10 mg/1 mL	40	187	0.16
NG2	5 mg/1 mL	20	210	0.49
NG3	5 mg/2 mL	40	133	0.14

**Table 2:** Summary of physico-chemical properties of PG-DOX-PTX NGs and controls.

#	Name	d [nm] Water	PDI	w% DOX	w% PTX	w% FITC	w% IDCC
**NG3**	PG-DOX NG	133	0.14	1.0	-		
**NG4**	PG-DOX_1_-PTX_1_ NG	148	0.12	0.5	0.9		
**NG5**	PG-PTX NG	148	0.23	-	1.9		
**NG6**	PG-DOX_5_-PTX_1_ NG	110	0.21	0.8	0.3		
**NG7**	PG-FITC NG	125	0.18	-	-	0.3	-
**NG8**	PG-DOX-IDCC NG	164	0.30	0.3	-	-	0.1

## Supplementary Materials

The following are available online at http://www.mdpi.com/2073-4360/10/4/398/s1, Table S1: PG-DOX-PTX NGs prepared with PG-DOX-PTX-SH conjugate as precursor and at different conditions, Figure S1: ^1^H-NMR spectrum (DMSO-*d*_6_) of macromonomer PG-PTX-SH (10%) conjugate, Figure S2: (a) Calibration curve for PTX in acetonitrile measured by RP-HPLC at a retention time of 2.85 min with acetonitrile-water (65:35) as mobile phase at a flow rate of 1.0 mL min^−1^ under isocratic regime. The injection volume was 50 μL. (b) Representative release profile of PG-PTX-DOX NG at pH 4.0 and 7.4 at 37 °C over 25 h. The PTX release (%) was quantified by RP-HPLC. Mean ± SEM were obtained from triplicates in three independent experiments, Figure S3: GPC chromatogram of NG3 at 480 nm, Figure S4: Representative release profile of PG-DOX NG at pH 4.0 and 7.4 over 45 h and calculated from the fluorescence spectra (Figure 8a,b) at 592 nm. The fluorescence intensity value at 45 h was set to 100% and the value at time point 0 to 0%, Figure S5: Fluorescence emission spectra of PG-DOX-SH conjugate after incubation in (a) acetate buffer (pH 4.0, 50 mM) and (b) phosphate buffer (pH 7.4, 50 mM) at 37 °C over 45 h.
