# Supplementary material for: A Facile, One-Pot, Surfactant-Free Nanoprecipitation Method for the Preparation of Nanogels from Polyglycerol–Drug Conjugates that Can Be Freely Assembled for Combination Therapy Applications
Source: Polymers (Basel). 2018 Apr 3;10(4):398. doi: 10.3390/polym10040398 (PMC6415236; doi:10.3390/polym10040398)
Supplement: Supplementary file 1 [file polymers-10-00398-s001.pdf]

Supporting Information for

A Facile, One-Pot, Surfactant-Free Nanoprecipitation Method for the Preparation of Nanogels from Polyglycerol–Drug Conjugates that Can Be Freely Assembled for Combination Therapy Applications

Laura I. Vossen, Stefanie Wedepohl and Marcelo Calderón *

Freie Universität Berlin, Institut für Chemie und Biochemie, Takustrasse 3, 14195 Berlin, Germany; laura.vossen@fu-berlin.de (L.I.V.); stefanie.wedepohl@fu-berlin.de (S.W.)

* Correspondence: marcelo.calderon@fu-berlin.de; Tel.: 49-30-838-59-368

Table S1. PG-DOX-PTX NGs prepared with PG-DOX-PTX-SH conjugate as precursor and at different conditions.

	c [monomers]	Acetone (mL)	d [nm] water	PDI
**PG-DOX-PTX NG**	10 mg/ 1 mL	40	262	0.10
**PG-DOX-PTX NG**	5 mg/ 1 mL	20	227	0.39
**PG-DOX-PTX NG**	5 mg/ 2 mL	40	221	0.33

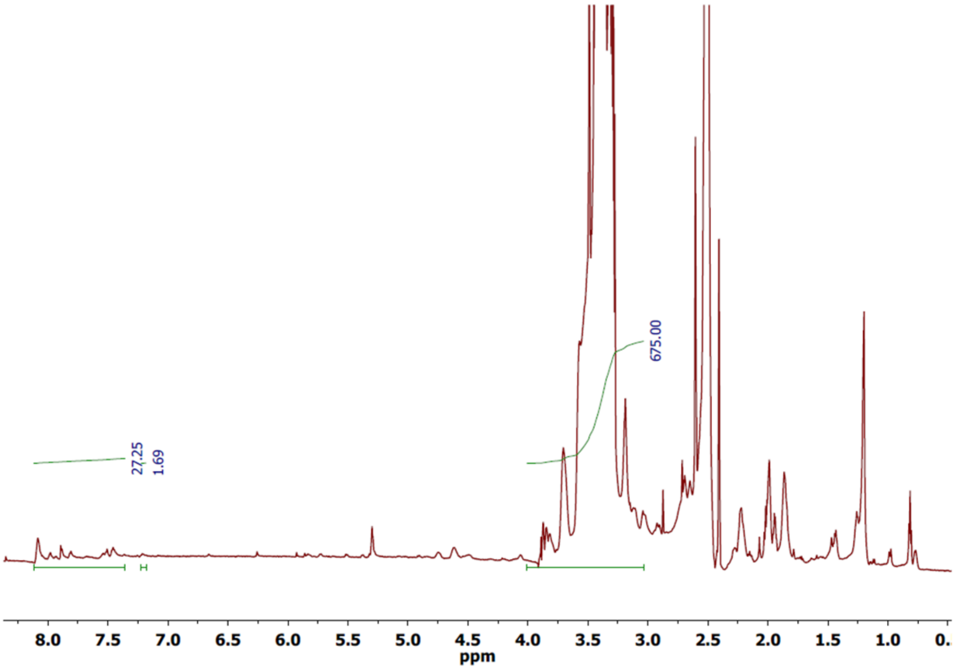

Figure S1. ^{1}H-NMR spectrum (DMSO-d_6) of macromonomer PG-PTX-SH (10%) conjugate.

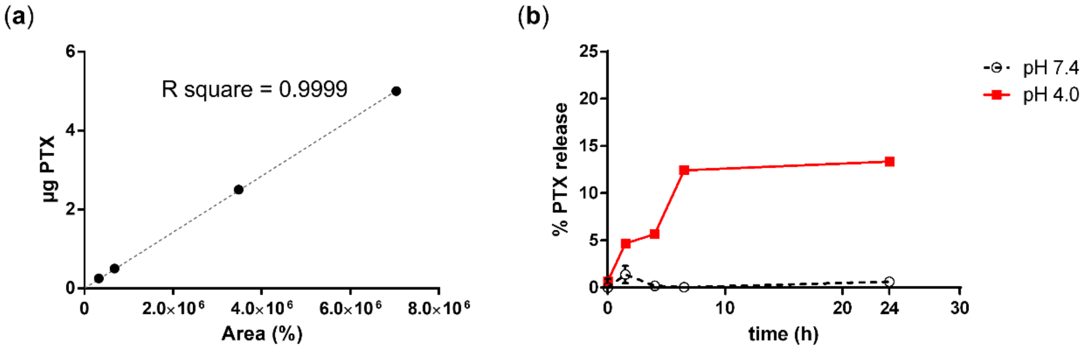

Figure S2. (a) Calibration curve for PTX in acetonitrile measured by RP-HPLC at a retention time of 2.85 min with acetonitrile-water (65:35) as mobile phase at a flow rate of 1.0 mL min^{-1} under isocratic regime. The injection volume was 50 µL. (b) Representative release profile of PG-PTX-DOX NG at pH 4.0 and 7.4 at 37 °C over 25 h. The PTX release (%) was quantified by RP-HPLC. Mean ± SEM were obtained from triplicates in three independent experiments.

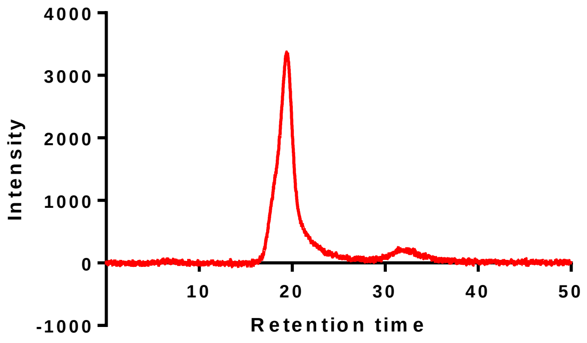

Figure S3. GPC chromatogram of NG3 at 480 nm.

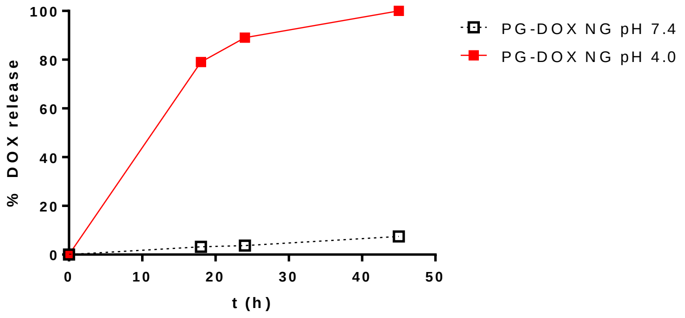

Figure S4. Representative release profile of PG-DOX NG at pH 4.0 and 7.4 over 45 h and calculated from the fluorescence spectra (Figure 8a and b) at 592 nm. The fluorescence intensity value at 45 h was set to 100% and the value at time point 0 to 0%.

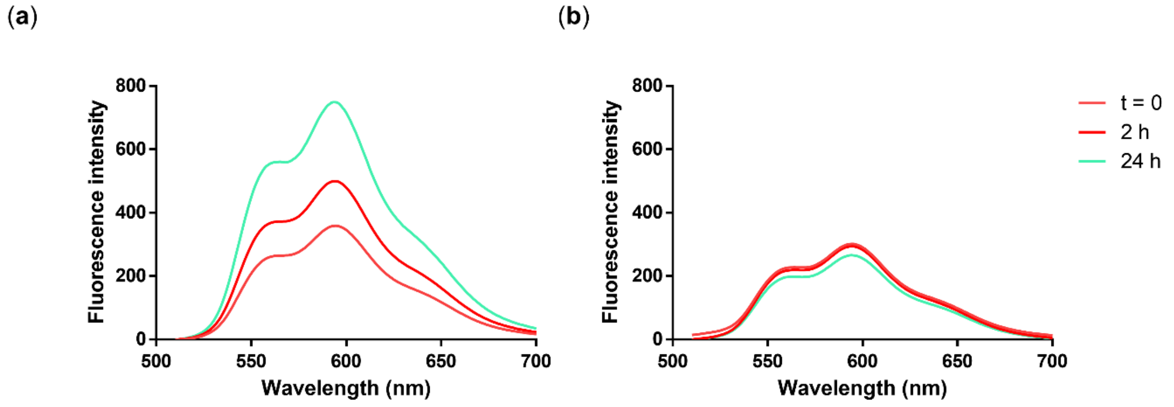

Figure S5. Fluorescence emission spectra of PG-DOX-SH conjugate after incubation in (a) acetate buffer (pH 4.0, 50 mM) and (b) phosphate buffer (pH 7.4, 50 mM) at 37 °C over 24 h.
